# CC-Chemokine receptor CCR7: a key molecule for lymph node metastasis in esophageal squamous cell carcinoma

**DOI:** 10.1186/1471-2407-14-291

**Published:** 2014-04-26

**Authors:** Tomoyuki Irino, Hiroya Takeuchi, Sachiko Matsuda, Yoshiro Saikawa, Hirofumi Kawakubo, Norihito Wada, Tsunehiro Takahashi, Rieko Nakamura, Kazumasa Fukuda, Tai Omori, Yuko Kitagawa

**Affiliations:** 1 Department of Surgery, Keio University School of Medicine, 35, Shinanomachi, Shinjuku-ku Tokyo 160-8582, Japan

**Keywords:** Chemokine, CCL21, Chemokine receptor, CCR7, Esophageal squamous cell carcinoma

## Abstract

**Background:**

CC-chemokine receptor 7 (CCR7), a known lymph node homing receptor for immune cells, has been reported as a key molecule in lymph node metastasis. We hypothesized a clinicopathological correlation and functional causality between CCR7 expression and lymph node metastasis in patients with esophageal squamous cell carcinoma (ESCC).

**Methods:**

We performed immunohistochemical analysis of 105 consecutive and 61 exclusive pathological T1 ESCC patients, followed by adhesion assay and *in vivo* experiment using a newly developed lymph node metastasis mouse model. The adhesive ability in response to CC-chemokine ligand 21/secondary lymphoid-tissue chemokine (CCL21/SLC) was assessed in the presence or absence of lymphatic endothelial cells and anti-CCR7 antibody. We established a heterotopic transplantation mouse model and analyzed lymph node metastasis by quantitative real-time RT-PCR.

**Results:**

Positive CCR7 expression in immunohistochemistory was detected in 28 (27%) of 105 consecutive patients and 17 (28%) of 61 T1 patients, which significantly correlated with lymph node metastasis (*p* = 0.037 and *p* = 0.040, respectively) and poor five-year survival (*p* = 0.013 and *p* = 0.012, respectively). Adhesion assay revealed an enhanced adhesive ability of CCR7-expressing cells in response to CCL21/SLC, in particular, in the presence of lymphatic endothelial cells (*p* = 0.005). In the mouse model, lymph nodes from mice transplanted with CCR7-expressing cells showed significantly higher DNA levels at 5 weeks (*p* = 0.019), indicating a high metastatic potential of CCR7-expressing cells.

**Conclusion:**

These results demonstrated the significant clinicopathological relationship and functional causality between CCR7 expression and lymph node metastasis in ESCC patients.

## Background

Esophageal cancer is one of the most malignant solid tumors with a five-year survival rate estimated at <19% [1]. Data reported by the International Union Against Cancer/ American Joint Committee on Cancer (UICC/AJCC) showed that approximately 44% of initially resected cancers of the esophagus and esophagogastric junction had lymph node metastasis and this high incidence contributed to the causes of poor survival [2]. Nevertheless some surgeons, especially those in Japan, have been conducting extended lymphadenectomy to provide better prognosis, most prospective trials have failed to demonstrate its survival benefit and its clinical significance is now considered to be limited [3-6].

One potential approach that might provide a breakthrough in this situation is the clarification of the mechanism of lymph node metastasis. A report by Müller et al. yielded important and noteworthy findings [7]. They found that the interaction between a chemokine and its corresponding chemokine receptor had a critical role in organ-specific cancer metastasis and they suggested that the CC-chemokine receptor 7 (CCR7) and CC-chemokine ligand 21/secondary lymphoid-tissue chemokine (CCL21/SLC) axis might be responsible for lymph node metastasis. Chemokine receptor CCR7 was first identified as a gene receptor of the Epstein–Barr virus (EBV), while CCL21/SLC and CC-chemokine ligand 19/ EBV-induced molecule ligand chemokine (CCL19/ELC) have since then been reported to be specific ligands for CCR7 [8]. CCR7 has been shown to be a homing receptor that controls the migration of immune cells to secondary lymphoid tissue organs in response to CCL21/SLC. Müller et al. demonstrated that this cell homing mechanism applied equally to CCR7-expressing cancer cells, allowing lymph node metastasis to be established as a consequence.

Although CCR7 and clinical associations have been revealed in a large variety of malignant tumors [9], only a few are currently known for esophageal cancer. In addition, little is known about the *in vitro* and *in vivo* behavior of CCR7-expressing cells in the sequence of lymph node metastasis. In this study, we hypothesized that patients with esophageal squamous cell carcinoma (ESCC) have a close relationship between CCR7 expression and lymph node metastasis because a functional CCR7 on ESCC cells plays a crucial role in lymph node metastasis in response to CCL21/SLC.

## Methods

### Study patients

We retrospectively surveyed ESCC patients who underwent radical esophagectomy in Keio University hospital between 1997 and 2007. Of these patients, we selected 105 consecutive patients from 1997 to 2002 who were diagnosed with Stage IA-IIIC and 61 patients from 1997 to 2007 who were diagnosed with pathological T1 cancer (tumor invasion into the lamina propia or submucosa), according to the UICC staging system (7^th^ edition).

### Immunohistochemistry

CCR7 expression in tissue samples was assessed by immunohistochemistry (IHC) using the ENVISION + system (Dako, Glostrup, Denmark). A rabbit anti-human polyclonal IgG CCR7 antibody (1:100; Medical and Biological Laboratories, Aichi, Japan) was used as a primary antibody. Paraffin-embedded human normal spleen and tonsil tissues were used as positive controls for CCR7. Negative control sections were treated with a non-immunized rabbit immunoglobulin fraction (Dako) under equivalent conditions.

To assess the immunoreactivity, the sections were scored in terms of their proportion (score 0: -10%, 1; 10%–40%, 2: 40%–70%, 3: >70%) and intensity (score 0: none, 1: weak, 2: strong) by two investigators (T.I. and H.T.) who had no knowledge of clinicopathological factors. The total score was calculated by multiplying the two scores and they were defined as positive at ≥1.

### Esophageal cell line

We used 10 established ESCC cell lines from the TE series (TE-1, 4, 5, 6, 8, 9, 10, 11, 14, and 15) kindly provided by Dr. Nishihira (Tohoku University, Miyagi, Japan). Identity of each cell line was confirmed by short tandem repeat (STR) analysis (data not shown).

### RNA extraction and quantitative real-time RT-PCR

Total RNA from each of the 10 TE cell lines was extracted and analyzed by quantitative real-time RT-PCR using the 7300 Real Time PCR system (Applied BioSystems, Carlsbad, CA), TaqMan Gene Expression Master Mix (Applied BioSystems), and ready-to-use CCR7 primers (Assay ID: Hs99999080_m1; Applied BioSystems). Glyceraldehyde-3-phosphate dehydrogenase (GAPDH) was used as an internal control. We used human lymphocyte from a healthy donor as a positive control and distilled water without the template as a negative control. The relative quantity of CCR7 mRNA in 10 TE cell lines was calculated using ΔΔCt method in which TE1 expression level is defined as 1.

### Establishment of a stable CCR7-overexpressing cell line

CCR7 mRNA was extracted from TE8 and its full-length open reading frame (ORF) was amplified and inserted into the plasmid vector pFLAG-CMV-4 (Sigma Aldrich, St. Louis, MO). The plasmids were electroporated into *E.coli* 5DHα (Takara Bio Inc., Shiga, Japan) and transfected to TE4 cells using Lipofectamine 2000 Reagent (Invitrogen, Carlsbad, CA). We defined this stable transfectant as TE4^CCR7+^ in which CCR7 overexpression was confirmed by western blotting, cell enzyme-linked immunosorbent assay (ELISA), and real-time RT-PCR.

### Adhesion assay

TE4 and TE4^CCR7+^ cells were labeled with green 5-chloromethylfluorescein diacetate (CMFDA; Invitrogen). To investigate the effect of epithelial cells on cell adhesion, normal human lung lymphatic microvascular endothelial cells (HMVEC-LLy; Lonza Walkersville Inc., Walkersville, MD) were used in this assay, and HMVEC-LLy was labeled with a red fluorescent dye (CMPTX; Invitrogen). Chambers were confluent with a monolayer of HMVEC-LLy. We seeded 5 × 10^4^ cells/well TE4 or TE4^CCR7+^ and incubated for 10 min at 37°C with or without human recombinant CCL21/SLC (R&D Systems). A blocking assay was performed using mouse monoclonal anti-CCR7 antibody (10 μg/ml; R&D Systems), mouse monoclonal anti-intercellular adhesion molecule (ICAM)-1 (10 μg/ml; Abcam, Cambridge, England), or anti-ICAM-2 antibody (10 μg/ml; Abcam). The number of attached cells in five randomly-selected fields was semiautomatically counted using BIOREVO BZ-9000 microscope (Keyence, Osaka, Japan). These procedures were repeated at least three times.

### Development of lymph node metastasis model and quantification of DNA from tumor cells in lymph nodes

Our heterotopic transplantation mouse model of lymph node metastasis was developed as follows: 1) a subcutaneously growing tumor was established by injecting 1 × 10^7^ TE4 or TE4^CCR7+^ cells into five-week-old nude mice; 2) after the tumors had developed (4–6 weeks after injection), they were excised and a small piece (approximately 1 mm^3^) was transplanted into the right and left elbows of additional five-week-old nude mice (defined as the TE4 and TE4^CCR7+^ groups, respectively); 3) the accessory axillary lymph nodes were excised and examined at 3, 4, and 5 weeks after transplantation. Ten lymph nodes from five nude mice were examined each week, and DNA of each node was extracted.

We used a human Alu sequence to quantitatively assess lymph node metastasis. We quantified 90 ng of DNA from each lymph node using primers for the Alu sequence [10]. The primers were 5’-CGCCTGTAATCCCAGCACTTT-3’ (forward), 3’-CCCAGGCTGGAGTGCAGT-5’ (reverse), and 5’-FAMCGAGGCGGGCGGATCACCTBHQ1-3’ (TaqMan Probe). We used DNA extracted from TE4 cells as a positive control while that from the lymph nodes of a non-treated nude mouse was used as a negative control. The estimated number of cells was calculated based on a standard curve prepared prior to this experiment (data not shown, *r*^*2*^ = 0.998).

### Statistical analyses

For statistical analyses, Student’s t-test, Pearson’s χ^2^ test or Fisher’s exact probability test was used for assessing the correlation between CCR7 expression and clinicopathological characteristics. The Kaplan–Meier and log-rank test were used for survival analysis. Student’s t-test or Welch’s t-test with Bonferroni correction was used for analysis in adhesion assay. In the mouse model, the Mann–Whitney U test was used to calculate statistical significances of the estimated number of cells. All statistical procedures were performed using IBM SPSS Statistics (Version 19; IBM, Armonk, NY). A *p* value of less than 0.05 was considered statistically significant. This study was approved by the Institutional Review Board at Keio University School of Medicine (ref. 20–125).

## Results

### CCR7 expression and clinicopathological characteristics

**Figure 1:**
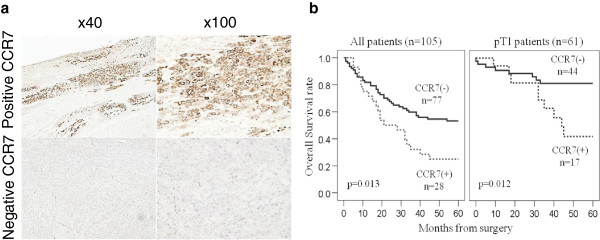
**Representative immunohistochemistry for CCR7 protein and the five-year overall survival rate in ESCC patients. a**. Representative immunohistochemistry for positive and negative expression of CCR7. **b**. Five-year overall survival rate in 105 consecutive patients and 61 exclusively T1 ESCC patients.

We found that 28 of 105 patients (26.7%) were positive for CCR7 expression (total score >1) and these were referred to as the CCR7(+) group (n = 28), whereas the remainder were the CCR7(-) group (n = 77) (Table 1). Representative images of IHC are shown in Figure 1a. The CCR7(+) patients exhibited a significantly higher rate of lymph node metastasis (*p* = 0.037) and a poor five-year overall survival (OS, 53.2% versus 25.0%, *p* = 0.013) (Figure 1b). We then tested whether the correlation was also applicable to early stage ESCC, because ESCC often leads to early lymph node metastasis when compared with other solid gastrointestinal cancers. We assessed 61 patients who were pathologically diagnosed with T1 cancer, with the result that 17 patients (27.9%) were classified in the CCR7(+) group (Table 1). The CCR7(+) group had significant correlations with lymph node metastasis (*p* = 0.040) and the histological grade (*p* = 0.020) as well as lymphatic and vessel invasions (*p* = 0.044 and *p* = 0.045, respectively). Patients with positive CCR7 expression also had a poor five-year OS (81.8% versus 47.1%, *p* = 0.012) (Figure 1b).

**Table 1:** CCR7 expression and clinicopathological characteristics of 105 consecutive patients and exclusively pT1 patients

	**All patients (n = 105) (%)**			**pT1 patients (n = 61) (%)**		
	**CCR7(+)**	**CCR7(-)**	**p**	**CCR7(+)**	**CCR7(-)**	***p***
	**n = 28 (27)**	**n = 77 (73)**		**n = 17 (28)**	**n = 44 (72)**	
Age (mean ± SD)	62.3 ± 8.5	59.2 ± 7.2	0.070^c^	59.9 ± 7.4	60.0 ± 7.6	0.995^c^
Gender						
Male	26	70	1.000^a^	15	39	1.000^a^
Female	2	7		2	5	
Histological grade						
1/2	26	69	1.000^a^	13	41	0.087^a^
3	2	8		4	3	
Pathological T						
T1/T2	16	41	0.894^b^	T1a 1	12	0.088^a^
T3/T4a	12	36		T1b 16	32	
Pathological N						
Negative	5	32	0.037^a^	6	30	0.040^b^
Positive	23	45		11	14	
UICC/AJCC stage						
I/II	11	41	0.296^b^	14	31	0.519^a^
III	17	36		3	13	
Lymphatic invasion						
Absent	2	16	0.144^a^	4	24	0.044^a^
Present	26	61		13	20	
Vessel invasion						
Absent	10	44	0.085^b^	10	38	0.045^b^
Present	18	33		7	6	

SD: standard deviation.

^a^: Fisher’s exact probability test, ^b^: Pearson’s χ-square test, ^c^: Student’s t-test.

Table 2 indicates the distribution of the total score of CCR7 expression. The number of patients with lymph node metastasis did not increase as the score increased. Thus, no apparent relationship between intensity/proportion of CCR7 expression and lymph node metastasis was found.

**Table 2:** Distribution of immunohistochemical CCR7 score and lymph node involvement

**All patients**								
CCR7 score	0	1	2	3	4	5	6	Total
No. of patients	77	2	8	1	8	0	9	105
LN (+)	45	2	7	1	5	0	8	68
LN (-)	32	0	1	0	3	0	1	37
**pT1 patients**								
CCR7 score	0	1	2	3	4	5	6	Total
No. of patients	44	1	5	0	9	0	2	61
LN (+)	13	1	3	0	5	0	2	24
LN (-)	31	0	2	0	4	0	0	37

LN: lymph node.

### CCR7 mRNA expression in TE cell lines

**Figure 2:**
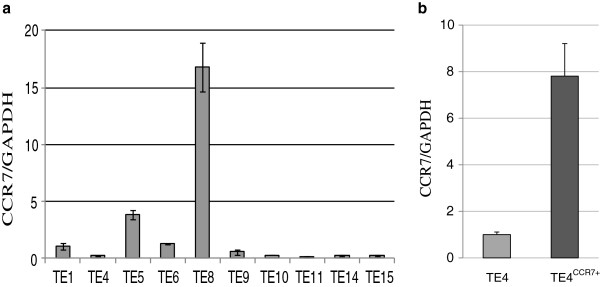
**CCR7 mRNA expression in TE cell lines by quantitative real-time RT-PCR. a**. CCR7 mRNA expression in 10 ESCC cell lines. Relative CCR7 mRNA levels were normalized with GAPDH and calculated using the ddCt method where TE1 was defined as 1. **b**. CCR7 mRNA expression in TE4 and TE4CCR7+. (*CCR7+ should be written in superscript letter).

CCR7 mRNA expression in TE cell lines was assessed by quantitative real time RT-PCR. (Figure 2a) The CCR7 mRNA expression level was extremely low compared with human lymphocytes (1.323 × 10^5^), whereas all TE cell lines expressed CCR7 at different relative copy levels ranging from 0.215 to 16.8 (median 0.43). This large difference among the cell lines was also seen in other studies in which the level of CCR7 expression of another ESCC cell line or other cancer cell lines was investigated [7,11,12]. TE4, TE10, TE11, TE14, and TE15 showed the lowest CCR7 mRNA levels and we selected TE4 for subsequent experiments because TE4 was the only cell line transplantable to mice among the five cell lines (data not shown). Then we established 18 TE4^CCR7+^ cell lines that overexpressed CCR7 by gene transfer and used one of the cell lines for further investigation which was transplantable to mice and showed 7.8-fold high CCR7 expression compared with untreated TE4 (Figure 2b).

### Effect of CCL21/SLC on adhesive ability

**Figure 3:**
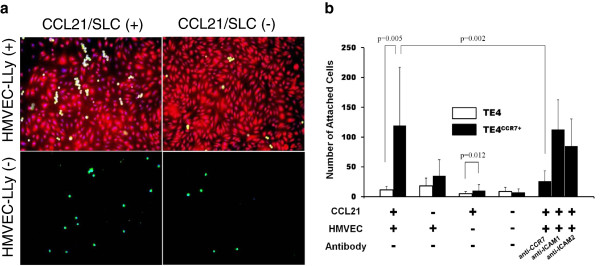
**Adhesion assay of TE4 and TE4**^**CCR7+ **^**cells. a**. Representative adhesion assay for TE4^CCR7+^ cells with or without CCL21/SLC. TE4^CCR7+^ cells were dyed using CMFDA (green), while lymphatic endothelial cells (HMVEC-LLy) were dyed using CMPTX (red). **b**. Number of attached cells in each experiment.

Figure 3a shows the effect of CCL21/SLC on cell adhesion, while Figure 3b presents a summary of the number of attached cells. CCL21/SLC significantly increased the number of attached TE4^CCR7+^cells, although not with TE4 cells in the presence of HMVEC-LLy (mean number ± SD (cells): 119.4 ± 97.6 versus 7.3 ± 5.6, *p* = 0.005). TE4^CCR7+^ cells lost their adhesive abilities when anti-CCR7 antibody was added (119.4 ± 97.6 versus 25.9 ± 17.7, *p* = 0.002). The CCL21/SLC facilitated cell adhesion in the presence of HMVEC-LLy, but only a small difference was found between the two cell lines in its absence (10.3 ± 9.7 versus 3.0 ± 2.1, *p* = 0.012). These results suggest that CCL21/SCL enhanced the adhesive ability of CCR7-expressing cells, especially in the presence of lymphatic endothelial cells. We also investigated whether ICAM-1 or ICAM-2 was involved in this adhesion mechanism by adding anti-ICAM-1 and anti-ICAM-2 antibodies, but we found no significant differences before and after the treatment.

### Lymph node metastasis in vivo

**Figure 4:**
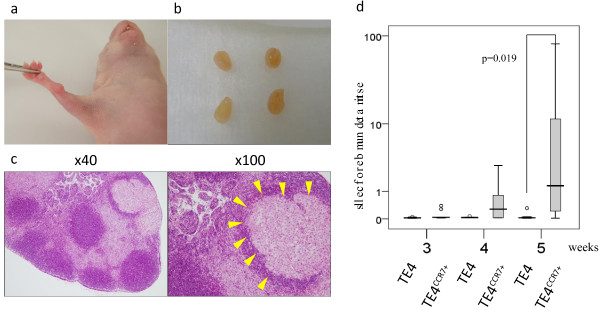
**Heterotopic transplantation mouse model for lymph node metastasis. a**. A subcutaneously growing tumor was excised and a small piece was transplanted into the elbow of another nude mouse. **b**. Gross appearance of excised accessory lymph nodes. **c**. Hematoxylin and Eosin staining of the excised lymph node (yellow arrow indicates metastatic cancer cells). **d**. Estimated number of cells in the accessory axillary lymph nodes, as measured by quantitative real-time RT-PCR with the human Alu sequence.

We established a heterotopic transplantation mouse model to investigate the metastatic ability of CCR7-expressing cells *in vivo* (Figure 4a). Although the excised accessory lymph nodes were grossly normal (Figure 4b), metastatic cells were demonstrated in the lymph nodes by Hematoxylin and eosin staining (Figure 4c). Figure 4d shows the estimated number of metastatic cells calculated from the quantitative DNA levels detected in the excised lymph nodes at 3, 4, and 5 weeks after transplantation. No differences were observed at 3 and 4 weeks after transplantation between the TE4^CCR7+^ and TE4 groups, but the TE4^CCR7+^ group showed significantly higher DNA levels at 5 weeks (mean ± SD (estimated number of cells): 15.09 ± 32.93 versus 0.11 ± 0.15, *p* = 0.019), indicating the high metastatic potential of CCR7-expressing cells.

## Discussion

Our study had three significant findings about CCR7 and lymph node metastasis in ESCC. First, we demonstrated that positive CCR7 expression was significantly correlated with lymph node metastasis and the five-year survival rate using the largest population of patients with ESCC. In addition, we also showed that exclusive pT1 patients denoted the same tendency. Second, the adhesive abilities of CCR7-expressing cells were significantly enhanced in response to CCL21/SLC, which has never been reported in prior studies. Third, CCR7-expressing cells had higher metastatic potential in the lymph node of our heterotopic transplantation mouse model, which was a newly-developed model in the study of lymph node metastasis.

Previous studies have described clinical correlations between CCR7 and lymph node metastasis in malignant solid tumors [7,13-15]. However, only three studies have investigated esophageal cancer [12,16,17]. Our results confirmed the results obtained in these previous studies, but with a larger population of patients and we provided further evidence of the correlation with ESCC. Another key finding was that patients with pathological T1 ESCC denoted the same tendency. This fact has rarely been described because of low incidence of lymph node metastasis in patients at an early stage of other carcinomas, whereas it can occur in 25%–35% of T1 ESCC patients [18]. These relationships between positive CCR7 expression and frequent lymph node metastasis imply the possible functional correlation of CCR7 with lymph node metastasis in ESCC.

Lymphatic invasion seems to have a significant relationship with positive CCR7 since CCR7 expression significantly correlated with lymph node metastasis, but there was no significant difference in lymphatic invasion when all patients with ESCC were analyzed. One possible reason for this is that lymphatic invasion was seen in most of patients with ESCC (83%), which, if any, required us more patients to be analyzed to demonstrate a significant difference. However, patients with both positive CCR7 and lymphatic invasion accounted for 93% of all patients with positive CCR7. Moreover, we showed significant differences in lymphatic and vessel invasion in pT1 patient population. These facts suggest a potential relationship between CCR7 expression and lymphatic invasion.

The adhesion assay demonstrated an enhanced adhesive ability of CCR7-expressing cells in response to CCL21/SLC, especially in the presence of lymphatic endothelial cells. Yin et al. investigated the functional and genetic importance of heparan sulfate lining on the lymphatic endothelial cells, demonstrating that heparan sulfate plays a critical role in mediating chemokine-dependent tumor cell trafficking in the lymphatic microenvironment [19]. Another study by Peramo et al. showed that heparan sulfate is a preferred substrate for adhesion while it is attenuated by heparin use [20]. Taken together, CCR7-mediated cell adhesion may be facilitated in the presence of lymphatic endothelial cells, presumably through heparan sulfate glycosaminoglycans. For naïve T cells, subsequent firm binding was established by ICAM-1 and ICAM-2; however, for cancer cells, this binding could not be blocked by anti-ICAM-1 and ICAM-2 antibodies. Further study will be required to clarify which molecule is involved in this firm binding to lymphatic cells since it can be a potential target for preventing cancer cell adhesion as well.

Transferring these findings to clinical practice demands a clinically accurate and reliable animal model. We demonstrated the high metastatic potential of CCR7-expressing cells using a heterotopic transplantation mouse model. With regard to animal models, Cunningham et al. and Wiley et al. have also reported the high metastatic potential of CCR7-expressing cells for breast cancer and malignant melanoma metastasis, respectively [21,22]. Our results objectively demonstrated not only the high metastatic potential of CCR7-expressing cells but also an earlier metastasis compared with wild-type cells. This result was comparable with that of our clinical study of T1 ESCC. Therefore, this may provide a molecular explanation for early lymph node metastasis in ESCC patients.

Our findings suggest potential therapeutic strategies for lymph node metastasis, i.e., CCR7 may be a promising biomarker and molecular target for lymph node metastasis. First, CCR7 could be a candidate that can predict lymph node metastasis. T1 patients with CCR7-negative ESCC may be at a low risk of lymph node metastasis; therefore, they could receive less invasive therapy, thereby avoiding radical esophagectomy. Second, in terms of molecular therapy, Lanati et al. showed Chemotrap-1, an engineered CCL21-soluble inhibitor that could block the chemokine-induced migration of cancer cells, reduced lymphatic invasion, tracking, and metastasis *in vivo*[23]. Taken together, our results and this evidence of an inhibitory molecule could bring CCR7-targeted therapy closer to clinical reality. Our findings provide further evidence for the mechanism of CCR7-mediated lymph node metastasis, leading to novel therapeutic strategies for regulating lymph node metastasis.

## Conclusions

In this study, we demonstrated the significant clinicopathological relationship and functional causality between CCR7 expression and lymph node metastasis in ESCC patients. CCR7 expression significantly correlated with poor outcomes in ESCC patients.

The adhesive abilities of CCR7-expressing cells were significantly enhanced in response to CCL21/SLC. CCR7-expressing cells had higher metastatic potential in the lymph node of our heterotopic transplantation mouse model, which was a newly-developed model in the study of lymph node metastasis.

## Abbreviations

CCL19/ELC: CC-chemokine 19/Epstein Barr virus-induced molecule ligand chemokine; CCL21/SLC: CC-chemokine ligand 21/secondary lymphoid-tissue chemokine; CCR7: CC-chemokine receptor 7; CMFDA: 5-chloromethylfluorescein diacetate; EBV: Epstein-Barr virus; ESCC: Esophageal squamous cell carcinoma; HEV: High endothelial venule; HMVEC-LLy: Normal human lung lymphatic microvascular endothelial cell; HRP: Horseradish hydrogen peroxide; ICAM: Intercellular adhesion molecule.

## Competing interests

All of the authors declare no competing interests.

## Author’s contributions

IT collected data of ESCC patients, carried out all experiments, wrote this paper and discussed results; TH conceived the study and supervised all experiments performed; MS supported and carried out all experiments performed; TH and KY supervised the study as principal investigator. All participants contributed commentary on and corrected the manuscript. All authors read and approved the final manuscript.

## Pre-publication history

The pre-publication history for this paper can be accessed here:

http://www.biomedcentral.com/1471-2407/14/291/prepub

## References

[B1] Jemal A Siegel R Xu J Ward E Cancer statistics, 2010 CA Cancer J Clin 2010 60 5 277 300 10.3322/caac.20073 20610543

[B2] Rice TW Rusch VW Ishwaran H Blackstone EH Cancer of the esophagus and esophagogastric junction: data-driven staging for the seventh edition of the American Joint Committee on Cancer/International Union Against Cancer Cancer Staging Manuals Cancer 2010 116 16 3763 3773 10.1002/cncr.25146 20564099

[B3] Omloo JM Lagarde SM Hulscher JB Reitsma JB Fockens P van Dekken H Ten Kate FJ Obertop H Tilanus HW van Lanschot JJ Extended transthoracic resection compared with limited transhiatal resection for adenocarcinoma of the mid/distal esophagus: five-year survival of a randomized clinical trial Ann Surg 2007 246 6 992 1000 discussion 1000–1001 10.1097/SLA.0b013e31815c4037 18043101

[B4] Hulscher JB van Sandick JW de Boer AG Wijnhoven BP Tijssen JG Fockens P Stalmeier PF ten Kate FJ van Dekken H Obertop H Tilanus HW van Lanschot JJ Extended transthoracic resection compared with limited transhiatal resection for adenocarcinoma of the esophagus N Engl J Med 2002 347 21 1662 1669 10.1056/NEJMoa022343 12444180

[B5] Grotenhuis BA van Heijl M Zehetner J Moons J Wijnhoven BP van Berge Henegouwen MI Tilanus HW DeMeester TR Lerut T van Lanschot JJ Surgical management of submucosal esophageal cancer: extended or regional lymphadenectomy? Ann Surg 2010 252 5 823 830 10.1097/SLA.0b013e3181fcd730 21037438

[B6] Nishihira T Hirayama K Mori S A prospective randomized trial of extended cervical and superior mediastinal lymphadenectomy for carcinoma of the thoracic esophagus Am J Surg 1998 175 1 47 51 10.1016/S0002-9610(97)00227-4 9445239

[B7] Muller A Homey B Soto H Ge N Catron D Buchanan ME McClanahan T Murphy E Yuan W Wagner SN Barrerak JL Mohark A Verastegui E Zlotnik A Involvement of chemokine receptors in breast cancer metastasis Nature 2001 410 6824 50 56 10.1038/35065016 11242036

[B8] Birkenbach M Josefsen K Yalamanchili R Lenoir G Kieff E Epstein-Barr virus-induced genes: first lymphocyte-specific G protein-coupled peptide receptors J Virol 1993 67 4 2209 2220 8383238 10.1128/jvi.67.4.2209-2220.1993 PMC240341

[B9] Ben-Baruch A Organ selectivity in metastasis: regulation by chemokines and their receptors Clin Exp Metastasis 2008 25 4 345 356 10.1007/s10585-007-9097-3 17891505

[B10] Waye JS Presley LA Budowle B Shutler GG Fourney RM A simple and sensitive method for quantifying human genomic DNA in forensic specimen extracts Biotechniques 1989 7 8 852 855 2631790

[B11] Takeuchi H Fujimoto A Tanaka M Yamano T Hsueh E Hoon DS CCL21 chemokine regulates chemokine receptor CCR7 bearing malignant melanoma cells Clin Cancer Res 2004 10 7 2351 2358 10.1158/1078-0432.CCR-03-0195 15073111

[B12] Ding Y Shimada Y Maeda M Kawabe A Kaganoi J Komoto I Hashimoto Y Miyake M Hashida H Imamura M Association of CC chemokine receptor 7 with lymph node metastasis of esophageal squamous cell carcinoma Clin Cancer Res 2003 9 9 3406 3412 12960129

[B13] Nakata B Fukunaga S Noda E Amano R Yamada N Hirakawa K Chemokine receptor CCR7 expression correlates with lymph node metastasis in pancreatic cancer Oncology 2008 74 1–2 69 75 18544997 10.1159/000139126

[B14] Gunther K Leier J Henning G Dimmler A Weissbach R Hohenberger W Forster R Prediction of lymph node metastasis in colorectal carcinoma by expressionof chemokine receptor CCR7 Int J Cancer 2005 116 5 726 733 10.1002/ijc.21123 15828050

[B15] Mashino K Sadanaga N Yamaguchi H Tanaka F Ohta M Shibuta K Inoue H Mori M Expression of chemokine receptor CCR7 is associated with lymph node metastasis of gastric carcinoma Cancer Res 2002 62 10 2937 2941 12019175

[B16] Ishida K Iwahashi M Nakamori M Nakamura M Yokoyama S Iida T Naka T Nakamura Y Yamaue H High CCR7 mRNA expression of cancer cells is associated with lymph node involvement in patients with esophageal squamous cell carcinoma Int J Oncol 2009 34 4 915 922 19287948 10.3892/ijo_00000217

[B17] Song Y Wang Z Liu X Jiang W Shi M CCR7 and VEGF-C: molecular indicator of lymphatic metastatic recurrence in pN0 esophageal squamous cell carcinoma after Ivor-Lewis esophagectomy? Ann Surg Oncol 2012 19 11 3606 3612 10.1245/s10434-012-2419-y 22644515

[B18] Tachibana M Kinugasa S Shibakita M Tonomoto Y Hattori S Hyakudomi R Yoshimura H Dhar DK Nagasue N Surgical treatment of superficial esophageal cancer Langenbecks Arch Surg 2006 391 4 304 321 10.1007/s00423-006-0063-3 16830151

[B19] Yin X Truty J Lawrence R Johns SC Srinivasan RS Handel TM Fuster MM A critical role for lymphatic endothelial heparan sulfate in lymph node metastasis Mol Cancer 2010 9 316 10.1186/1476-4598-9-316 21172016 PMC3019167

[B20] Peramo A Meads MB Dalton WS Matthews WG Static adhesion of cancer cells to glass surfaces coated with glycosaminoglycans Colloids Surf B: Biointerfaces 2008 67 1 140 144 10.1016/j.colsurfb.2008.07.019 18815015

[B21] Cunningham HD Shannon LA Calloway PA Fassold BC Dunwiddie I Vielhauer G Zhang M Vines CM Expression of the C-C chemokine receptor 7 mediates metastasis of breast cancer to the lymph nodes in mice Transl Oncol 2010 3 6 354 361 10.1593/tlo.10178 21151474 PMC3000460

[B22] Wiley HE Gonzalez EB Maki W Wu MT Hwang ST Expression of CC chemokine receptor-7 and regional lymph node metastasis of B16 murine melanoma J Natl Cancer Inst 2001 93 21 1638 1643 10.1093/jnci/93.21.1638 11698568

[B23] Lanati S Dunn DB Roussigne M Emmett MS Carriere V Jullien D Budge J Fryer J Erard M Cailler F Girard JP Bates DO Chemotrap-1: an engineered soluble receptor that blocks chemokine-induced migration of metastatic cancer cells in vivo Cancer Res 2010 70 20 8138 8148 10.1158/0008-5472.CAN-10-0175 20736366 PMC3034641

